# Effects of Korean red ginseng (*Panax Ginseng Meyer*) on bisphenol A exposure and gynecologic complaints: single blind, randomized clinical trial of efficacy and safety

**DOI:** 10.1186/1472-6882-14-265

**Published:** 2014-07-25

**Authors:** Mihi Yang, Ho-Sun Lee, Min-Woo Hwang, Mirim Jin

**Affiliations:** Research Center for Cell Fate Control, College of Pharmacy, Sookmyung Women’s University, Hyochangwon-gil 52, Yongsan-Gu, Seoul, 140-742 Republic of Korea; Department of Sasang Constitutional Medicine, College of Korean Medicine, Kyung Hee University, Seoul, Republic of Korea; Labobatory of Pathology, College of Oriental Medicine, Daejeon University, Daejeon, Republic of Korea

**Keywords:** Korean red ginseng, Bisphenol A, Chemoprevention, Sasang typology, Gynecologic complaints, Quality of life

## Abstract

**Background:**

Korean red ginseng (KRG) is a processed ginseng from raw ginseng to enhance safety, preservation and efficacy, known having beneficial effects on women’s health due to its estrogen like function. While estrogen supplementation showed some modulation of endocrine disrupting chemicals, bisphenol A (BPA) has been focused as a potential endocrine disrupting chemical. In this study, we examined the efficacy and safety outcomes of KRG against BPA, focusing on female quality of life (QOL). Individual variations in susceptibility to KRG were also investigated with the Sasang Typology, the personalized medicine used for hundred years in Korea.

**Methods:**

We performed a single-blind randomized clinical trial. Study subjects were young women (N = 22), consumed 2.7 g of KRG or placebo per day for 2 weeks and filled up questionnaires regarding gynecologic complaints at the 4 time spots. We analyzed urinary total BPA and malondialdehyde (MDA), an oxidative stress biomarker, with GC/MS and HPLC/UVD respectively, and diagnosed their Sasang Typology with the questionnaire for the Sasang constitution Classification (QSCC II).

**Results:**

KRG consumption decreased urinary BPA and MDA levels (ps < 0.05) and alleviated ‘menstrual irregularity’, ‘menstrual pain’, and ‘constipation’ (ps < 0.05). SoEum type (Lesser Yin person) among the Sasang types showed significant alleviation in insomnia, flushing, perspiration and appetite by KRG consumption, rather than other Sasang types. During the intervention, no one experienced any aggravated side effects.

**Conclusion:**

We suggest KRG is efficient for protection for female QOL and BPA- exposure and – related oxidative stress. However, individual variation in susceptibility to KRG should be further considered for identifying ideal therapy.

**Trial registration:**

KCT0000920.

## Background

Ginseng (*Panax Ginseng Meyer*) is one of the highest selling herbal supplements in the world. The products of Korean red ginseng (KRG) have been sold over half of functional foods in Korea [1]. It costs approximately 600 million dollars in 2010. KRG has been traditionally obtained through a steaming process from raw ginseng to enhance safety, preservation and efficacy [2]. In the course of the steaming process, ginseng starch is gelatinized, causing an increase in saponin content [3] (Table 1). In addition, various functions of KRG have been reported, e.g. anti-inflammatory, −coagulating, and -oxidative actions, enhancement of sexual function, and vasodilation [4–8]. Moreover, some researchers predicted beneficial effects on KRG on women’s health, e.g. targeting postmenopausal symptoms, due to its estrogen like function [9, 10].

**Table 1:** **Comparison of ginsenoside contents (%) between Korean white and red ginseng** ^**3**^

Ginseng	Rg1	Re	Rf	Rh1	Rg2	Rb1	Rc	Rb2	Rd	Rg3
White	0.537	1.34	0.31	0.00	0.11	1.81	1.51	2.14	0.41	0.04
Red	0.492	1.11	0.24	**0.12**	0.13	1.96	1.47	2.17	**0.72**	**0.12**

**Bold**, higher in KRG than raw ginseng.

Estrogen supplementation showed modulation of an endocrine disrupting organochlorine, 3,3′,4,4′,5-pentachlorobiphenyl (PCB126) in rat bone and uterus [11]. Ginseng improved the survival rate and sperm quality in the guinea pigs exposed to 2,3,7,8-tetrachlorodibenzo-p-dioxin (TCDD), a notorious endocrine disrupting chemical (EDC) [12]. In addition, KRG extract activated both estrogen receptor (ER)α and ERβ and modulated the mRNA levels of estrogen-responsive genes, such as pS2 and ESR1 *in vitro* study, e.g. MCF-7 cells, however, estrogenic activity of KRG was not manifested *in vivo* study such as female Sprague–Dawley rats [13]. In human (women), KRG showed improvements in the Kupperman index and in the menopause rating scale scores [14]. Taken together, we can suspect effects of KRG on female reproduction or gynecological complaints via phytoestrogenic structures or characteristics of triterpenoid saponins in KRG.

However, ginsengs, usually white (raw) ginsengs, are stimulants and may cause nervousness or sleeplessness. There were some reports for their side effects, e.g. high blood pressure, insomnia, restlessness, anxiety, euphoria, diarrhea, vomiting, headache, nosebleed, breast pain or vaginal bleeding [15]. On the other hand, processed ginseng, i.e. KRG, have been broadly used with less toxicity after the process from raw ginseng. To avoid side effects of ginseng, ‘the Sasang (四象, four Typology or Constitution)’, which explains the individual differences in behavioral patterns, physical characteristics and susceptibility to a certain disease and medicinal herb based on their bio-psychological traits, has strongly recommended ginseng for Soeumin (少陰人, SE type) among the four types [16, 17].

**Figure 1:**
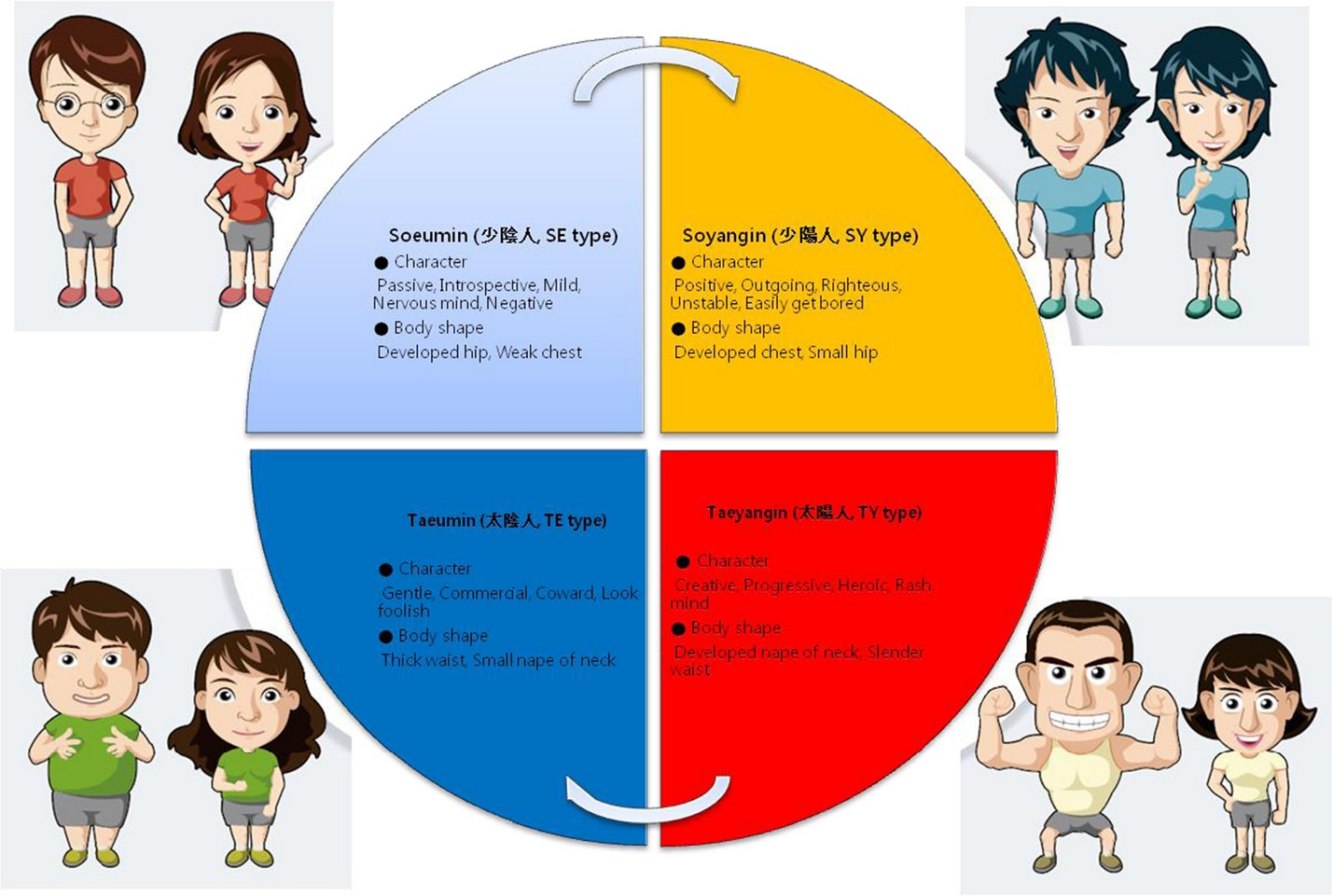
**Characteristics of the Sasang (四象, four types) constitutions.**

The Sasang medicine was systematically theorized by Jae-Ma Lee in his book ‘Dong-Yi-Soo-Se-Bo-Won (東醫壽世保元)’ [The Principle of Life Preservation in Oriental Medicine] published in the end of 19 century [16]. It shows temperament-based guideline for safe and effective use of medical herbs including ginseng, ginger, mahuang (*Ephedra Sinica*) and aconite, the latter two with fatal adverse effects. This guideline has been widely used by traditional Korean medicinal doctors clinically for over a century in Korea [18]. The four Sasang constitutions, Taeyangin (太陽人, TY type), Soyangin (少陽人, SY type), Taeeumin (太陰人, TE type), and Soeumin (少陰人, SE type), were distinguished according to individuals’ (1) sensitivity to certain groups of herbs and medicines, (2) equilibrium among internal organ functions, (3) physical features and (4) psychological characteristics [19] (Figure 1).

On the other hand, EDCs have been received attention among scholars and publics, because of their toxicities threatening fertility, intelligence and survival [20]. Among various EDCs, bisphenol A [2,2-bis(4-hydroxyphenyl)propane, BPA] has been focused as a potential EDC due to broad uses of BPA products, e.g. plastic bottles, containers, tooth sealing, etc. Therefore, people can be easily exposed to BPA in daily life. However, health risks of BPA are not clearly determined, yet. Metabolites of BPA, such as quinone- or semiqunone-compounds, are suspected as toxic materials to induce reactive oxygen species (ROS) [21]. ROS showed pathological roles in the female reproductive tract [22]. BPA was detected in the urine of the majority of women undergoing *in vitro* fertilization, and was inversely associated with number of oocytes retrieved and peak oestradiol levels [23]. We reported that BPA induces oxidative biomarkers, malondialdehyde (MDA) and 8-hydroxydeoxyguanosine [24]. Therefore, BPA itself or its induced ROS can be risky for female reproductive organs.

In this study, we investigated the chemopreventive effects of KRG against BPA and individual variations in susceptibility to KRG regarding female quality of life (QOL) to improve and to extend the application of KRG. The Sasang Typology was used for individual variation for susceptibility to the KRG.

## Methods

### Materials

**Figure 2:**
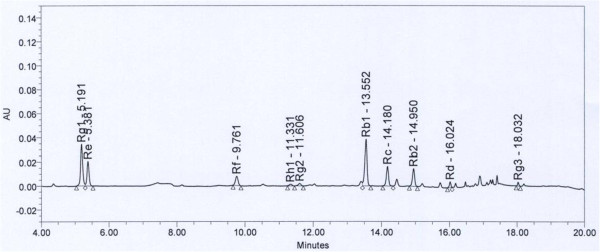
**The HPLC profile of KRG-capsule shows 10 major ginsenosides with detection time (KRG powder, 1.0037 g; dilution 19.93 folds; ginsenoside Rg1+ Rb1 = 6.87 mg/g).**

BPA, BPA-d16 and β-glucuronidase were purchased from Sigma Chemical Co. (St. Louis, MO). Methyltertialrybutylether (MTBE) was purchased from Burdick & Jackson (Muskegon, MI). N,O-Bis (trimethylsilyl) trifluoroacetamide (BSTFA) and trimethylchloro-silane (TMCS) were purchased from Supelco (Bellefonate, PA). KRG and placebo capsules were kindly provided by the Korean Ginseng Corp (Daejeon, S. Korea). These KRG capsules contained 300 mg of 6-year old KRG. Following quality control guidelines of Korean FDA [25], we obtained quality guaranteed KRG capsules with identified 10 ginsenosides including Rb1 and Rg1 from the Korean Ginseng Corp (Figure 2). Placebo capsules did not contain KRG but contained the same quantity of free-dried corn starch powder with the KRG capsules, i.e. 238.13 mg of corn starch, 10 mg of powder flavor of KRG and 1.88 mg of coloring matters.

### Clinical trial

This trial was designed as a single-blind randomized study, which was approved by Institutional Review Board of the Sookmyung Women’s university (SM-IRB-10-0720-004: Seoul, S. Korea). Inclusion criteria for the present study are healthy young women (21–30 yrs), however, who experienced pre-menstrual syndrome, e.g. menstrual pain, menstrual irregularity, etc. We excluded the people, who had any other health problems or took medicine. All participants filled out the informed consents before participating in this study. Volunteers who experienced menstrual pain or irregularity were recruited on bulletins in the above school. The consumption of KRG or placebo was determined by lot, a simple random sampling. Considering safety and other previous clinical trials [26, 27], we administered 9 capsules of placebo or KRG (2.7 g of KRG powder)/day to the subjects after meals for 2 weeks (N = 22: N = 11 for KRG; N = 11 for placebo). During the trial period, we collected their urine before breakfast on 4 spots, day 0, 4, 8 and 14 days and stored at −20°C until analysis was performed. At the same time, all subjects recorded daily food intake (items and volume/meal) during the trial and filled out questionnaires containing items of QOL, side effects of raw ginseng and potential endpoints of EDCs including the 17 complaints, i.e. menstrual pain, menstrual irregularity, cold hands and feet, anemia, dyspepsia, tiredness, loss of appetite, insomnia, diarrhea, constipation, face flushing, dipsosis, headache, palpitation, perspiration, nose -bleeding, and allergy, before the trial and after the next menstruation. These complaints were measured on 5-rating scales (1 to 5, severity increased with the rate, i.e. 1 = not at all; 5 = severely affected]. We asked the subjects whether they consumed KRG or placebo by daily phone calls and followed them up to 3 weeks after the trial.

In addition, we diagnosed all of the subjects into the four types of the Sasang constitution with the questionnaire for the Sasang constitution classification (QSCC) II made by the Sasang Medical Society of Korean Oriental Medical Society [28]. Reliability of the QSCC II was confirmed by diagnoses of Dr. Hwang M-W, an expert of the Sasang medicine. Based on our previous pharmacokinetic study among female subjects (N = 21) for 2 weeks regarding the intervention of KRG [2], we designed the present study with over 20 subjects.

The participants were compensated not for the benefit but for time spent and inconvenience associated with participation in research activities. The IRB approved the appropriateness of compensation.

### Analysis of urinary BPA

We analyzed urinary BPA with GC/MS, following Kim et al. method [29] with minor modification. In brief, the internal standard, BPA-d_16_, was added to 2 ml of each urine sample and 1 ml of 0.2 M sodium acetate buffer (pH 5.2) and 50 μL of 0.1% β-glucuronidase were added to the above urine samples. After adding 1 ml of 5% K^2^CO^3^ (pH 7), we incubated the reaction mixture at 55°C for 3 hrs and performed twice of liquid-liquid extraction with 2.5 ml of MTBE to collected supernatant after centrifuge. For derivatization, 50 μl BSTFA/TMCS (99:1 v/v) was added to the sample and the mixture was incubated in 60°C for 30 min. We applied 1 μl of the derivatized sample to GC/MS: Agilent 7890 plus gas chromatograph with a DB-5 ms (SW 54 bonded phase) capillary column (30 m × 0.25 mm I.D., 0.25 μm film thickness), interfaced to an Agilent 5975C mass selective detector (70 eV, electron impact mode). One μl of the each sample was injected in split ratio of 5:1. The flow rate was 9 ml/min. Helium was used as a carrier gas for 1.0 ml/min and the temperature of injector was 280°C. The oven temperature was initially 120°C for 1 min, raised to 320°C at 20°C/min, and held for 2 min. Selected Ion Monitoring (SIM) was performed for quantification of BPA at m/z 357 and internal standard BPA-d_16_ at m/z 368.

### Analysis of urinary MDA

We analyzed urinary MDA as an oxidative stress biomarker by its adducts with 2-thiobarbituric acid (TBA, CAS number: 504-17-6) as a previously described method [24] with minor modifications. Briefly, 300 μl of 0.5 M of phosphoric acid was mixed with 50 μl of urine or 1,1,3,3,-tetraethoxypropane (CAS number: 122-31-6) standards and 150 μl of TBA reagent. The mixtures were heated at 100°C for 1 h. The tubes were chilled on ice for 5 min. Five hundred μl of methanol was added to the mixtures, and the mixtures were centrifuged at 13,000 × g for 10 min. The supernatant fractions were transferred to glass autosampler vials and 20 μl of each supernatant was analyzed by HPLC. The levels of TBA-MDA adduct were determined at 532 nm on an isocratic HPLC system: The TSK gel column ODS-80TM (5 μm, 4.6 mm × 150 mm, Tosoh, Tokyo, Japan) was eluted with 50 mol/l potassium phosphate buffer (pH 6.8) and methanol (58:42, v/v). The flow rate of mobile phase was 0.6 ml/min.

The HPLC system was composed of dual Younglin SP930D pumps (Younglin, Seoul, Korea), MIDAS COOL autosampler (Spark Holland, Emmen, Netherlands) and SPD-10A UV–VIS detector (Shimadzu, Kyoto, Japan).

### Statistical analyses

To confirm no differences between KRG and placebo groups before the trial, we analyzed characteristics of subjects with t-test or Fisher exact test. Shapiro-Wilk W test was used to study the degree of normal distribution of BPA levels and the 17 pharmacodynamic parameters including menstrual pain, constipation, insomnia, etc. Regression analysis was used to study association between the KRG treatment and urinary BPA or MDA levels. Effects of KRG or BPA on the pharmacodynamics parameters were analyzed with t-test or Wilcoxon test due to the normality of the parameters. ANOVA (Analysis of variance) or Kruskal-Wallis test were also used to study effects of the Sasang Typology on responses to KRG.

All statistical analyses were performed with JMP (Version 4, SAS institute, Cary, NC). Statistical significance was defined as p < 0.05.

## Results

### Characteristics of subjects

All of the subjects were non-smoking women, and most of them experienced menstrual irregularity or menstrual pain [≥3 of the scale for disturbance in daily life (1 to 5), 1 = not at all; 5 = severely affected]. There were no significant differences in the characteristics of subjects between the KRG and placebo groups before the intervention (Table 2). In addition, there were no differences in the degree of the 17 symptoms between the two groups (ps > 0.05). Thus, we confirmed the confidence of the randomization in subjects for the intervention.

**Table 2:** **Characteristics of subjects before the trial**

General (mean ± std) ^a^	KRG (N = 11)	Placebo (N = 11)	p
Age (years)	22.91 ± 1.81	22.73 ± 1.68	0.81
BMI (kg/m^2^)	20.12 ± 1.69	20.83 ± 2.24	0.42
Urinary total BPA (ng/L)	3.17 ± 2.28	2.38 ± 1.98	0.41
**Diet** [N(%)]^b^			
Instant food intake^c^			1.00
High	1 (9)	0 (0)	
Moderate	4 (36)	4 (36)	
Low	6 (55)	7 (64)	
Diet balance^d^			1.00
Prefer meat	3 (27)	2 (18)	
Balanced	8 (73)	9 (82)	
Prefer vegetable	0 (0)	0 (0)	
**Sasang** [N(%)]^b^			
SY^e^	4 (36)	5 (45)	1.00
SE	3 (27)	2 (18)	
TE	4 (36)	4 (36)	

^a^T-test; ^b^Fisher exact test (2-tail).

^c^Degree of instant food-intake was classified with frequency: high, >10 times/week; moderate, 5–10 times/week; low, <5 times/week).

^d^Frequency (times) of meat or fish consumption/week, ≥10 times, prefer meat; 3–9 times, balanced; ≤2, prefer vegetable.

^e^Soyangin (少陽人, SY type); Soeumin (少陰人, SE type); Taeeumin (太陰人, TE type).

### Lack of association between BPA exposure and gynecologic complaints

**Figure 3:**
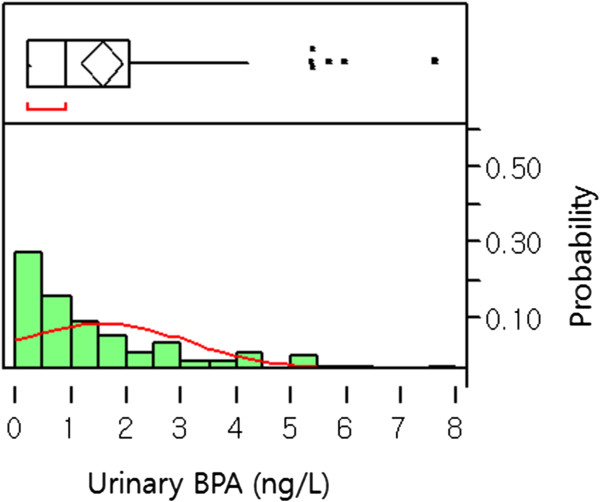
**Distribution of urinary BPA levels (ng/L): Histogram of urinary BPA levels: X and Y axes show levels of BPA (ng/L) and probability of all samples (N = 88 from 4 spots of 22 study subjects), respectively.** Upper part of figure shows an outlier box plot with the square in the box showing the interquartile range.

We analyzed levels of total BPA (free or conjugated BPA with phase II enzymes) in urine samples and used them as a biomarker for exposure monitoring of BPA. They were detected in 69% of the subjects during the trial. ‘BPA non-detectable’ samples were assigned 0.25 ng/L, half of the limit of quantification (LOQ = 0.5 ng/L) of analyzed BPA levels. The range of urinary total BPA was 0.25 ~ 7.60 (median 0.94) ng/L (Figure 3).

In addition, we analyzed associations between urinary total BPA levels before the trial, i.e. the 0 day-levels, and the 17 complaints, which might influence QOL in females and has been suspected as end points of EDCs. As results, we could not find any association between urinary total BPA levels and any complaint or the sum of complaints (ps > 0.05).

### Interaction between BPA and KRG

**Figure 4:**
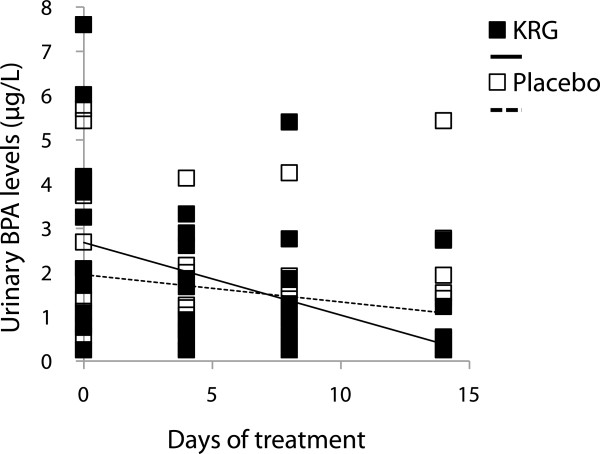
**KRG decreased urinary BPA levels: KRG group, r = −0.16, p < 0.01; placebo group, r = −0.06, p = 0.17 by regression analysis.**

The KRG treatment significantly decreased urinary BPA levels during the trial; however, the placebo did not (Figure 4). This trend was shown from the 4th day of the KRG treatment [levels of urinary BPA ‘before KRG treatment’ *vs.* ‘at the 4th day of the KRG’ intake: mean ± std, 3.16 ± 2.28 ug/L *vs.* 1.38 ± 1.14 ug/L: p = 0.03 by Wilcoxon test). Thus, effects of the KRG on urinary BPA levels could even be detected in 4 days after initiation of the KRG consumption.

As food can be the main exposure source of BPA [24], we let the subjects have their usual diet style and keep the same levels of BPA exposure during the intervention. Therefore, we could not find any significant change in urinary BPA levels in the placebo subjects during the intervention. Thus, the above inverse relationship between the KRG treatment and urinary BPA levels reflects that KRG protects from environmental BPA exposure via inhibition of its absorption. We also analyzed association between ‘KRG-reduced urinary BPA levels’ and ‘alleviation of the 17 complaints’ during the 2 weeks-trial. However, we could not find any significant association between them (ps > 0.05).

### Effects of KRG on urinary MDA

The range of urinary MDA levels was 0.37-6.7 uM (median, 2.34 uM). At first, we studied whether BPA induced urinary MDA or not and found positive association between urinary BPA and MDA levels (slope = 0.88, R2 = 0.10, p < 0.01). Therefore, we confirmed BPA induced urinary MDA in the present subjects.

Secondly, we investigated effects of KRG on BPA-induced MDA and found reduced urinary MDA levels not in the control (slope = −0.02, R2 = 0.01, p = 0.62) but in the KRG group (slope = −0.09, R2 = 0.11, p = 0.04). Therefore, we can estimate KRG has desirable effects on BPA-related oxidative stress.

### Effects of KRG on the 17 complaints

**Figure 5:**
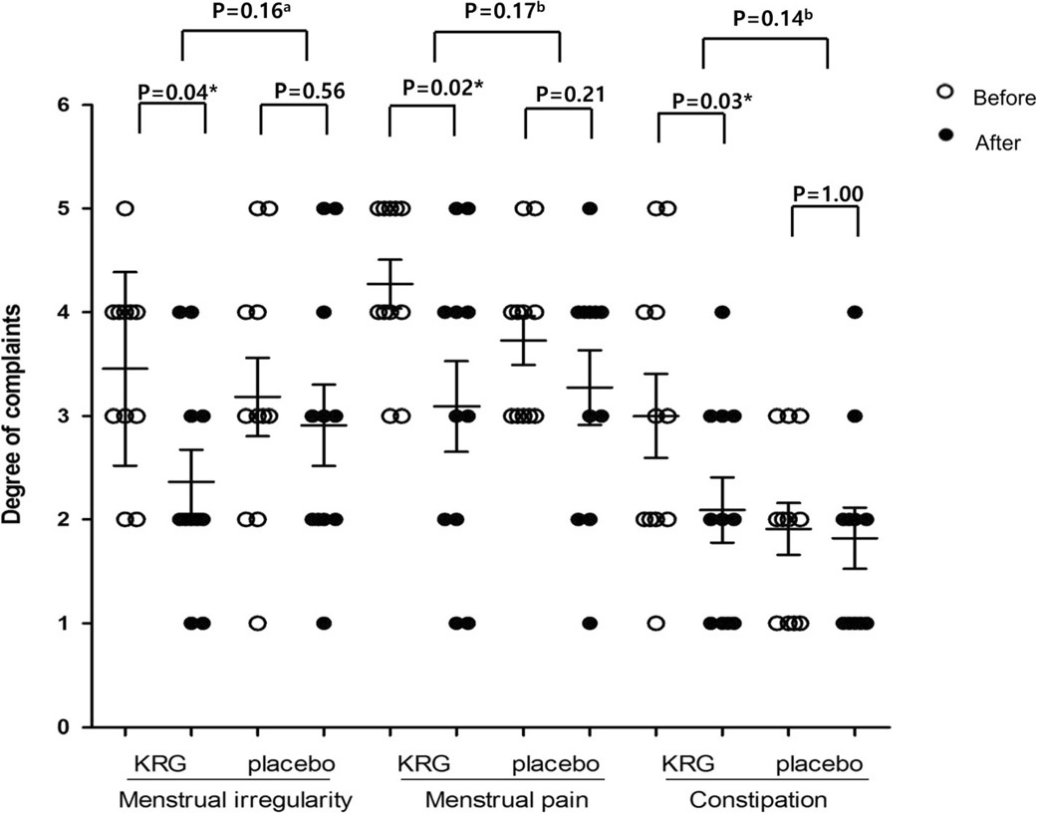
KRG lowered women’s gynecological and QOL-related complaints: *<0.05; ^**a**^ **student t-test;** ^**b**^ **Wilcoxon test.**

We studied effects of KRG on the 17 complaints. As results, we found that the KRG treatment significantly alleviated ‘menstrual irregularity’, ‘menstrual pain’, and ‘constipation’, although the above significances were not confirmed by the relative comparison with placebo (Figure 5).

In addition, there was no one who quit the trial due to side effects of KRG and no one experienced side effects of raw ginseng, e.g. insomnia, diarrhea, headache, nosebleed, breast pain, etc., in the present study. Therefore, KRG has a potential to be safe and chemopreventive for the above complaints.

### Effects of the Sasang constitutions on the KRG efficacy

**Figure 6:**
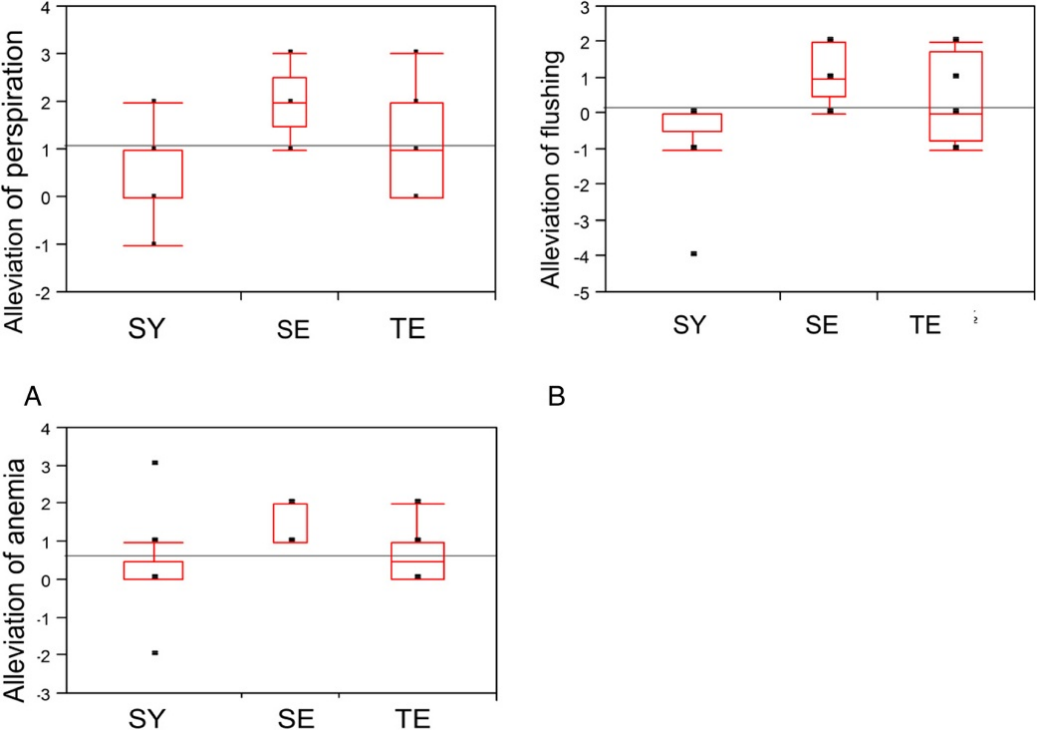
**The Sasang Constitutions showed different responses to KRG (N = 4, 3, and 4 for SY, SE and TE, respectively, in the KRG-treated group): A**. The Sasang Constitutions showed significantly different responses in KRG-induced alleviation of perspiration: p = 0.04 by ANOVA); **B**. The Sasang Constitutions showed significantly different responses in KRG-induced alleviation of flushing (p = 0.03 by Kruskal-Wallis test); **C**. The Sasang Constitutions showed significantly different responses in KRG-induced alleviation of anemia (p = 0.04 by Kruskal-Wallis test).

We diagnosed the Sasang constitutions of the subjects with the QSCC II. As a result, there were three kinds of constitutions in the present subjects: SY (N = 9), SE (N = 5) and TE (N = 8). Considering the Sasang constitutions, we analyzed effects of KRG on the 17 complaints. As results, susceptibility to KRG-induced alleviation of perspiration, anemia and flushing were different due to the Sasang constitutions (Figure 6): Particularly, the SE type showed sensitive responses to KRG, compared to other types. When we analyzed the differences of the KRG effects on the above three complains between two groups (SY vs. SE; SE vs. TE; SY vs. TE), we found borderline significant alleviation of all of the complains in the SE type, compared to that in the SY type (0.05 < ps < 0.1). However, there were no differences of the KRG effects between the SE and TE types and between the SY and TE types.

After regrouping the subjects into two groups, i.e. the SE type and the other (SY and TE) type, we observed appetite and insomnia in the 17 complains were significantly reduced by the KRG in the SE type, compared to the other types (ps < 0.05).

When we compared effects of KRG on all complains to those of placebo in the same Sasang type, we found insomnia in the SE type and headache and menstrual pain in the TE type were significantly reduced by the KRG consumption (ps < 0.05).

## Discussion

EDCs are toxic chemicals to target reproductive organs and induce infertility [30–32]. Titus-Ernstoff et al. provided evidence of menstrual irregularity and delayed menstrual regularization in the daughters of women exposed to diethylstilbestrol *in utero* [33]. Menstrual irregularity and pain among the studied 17 complaints are well known complaints, which influence QOL in females and are associated with various female reproduction diseases [34, 35]. BPA has been suspected to disrupt female reproduction systems: For example, levels of BPA in blood are associated with a variety of conditions in women including endometrial hyperplasia, recurrent miscarriages, sterility, and polycystic ovary syndrome [31]. However, some diet, such as folic acid, soy products, etc., can modify end points of BPA via epigenetic modulation [36]. Therefore, we studied effects of BPA on female reproductive complaints, however, could not find any association between exposure levels of BPA and female symptoms. Thus, current BPA exposure levels in the present study may not affect female complaints. In addition, simultaneous exposure of chemopreventive materials with BPA in real environment is suspected to alleviate BPA toxicity.

Secondly, we focused on effects of KRG on BPA exposure and found KRG lowered urinary BPA levels (Figure 4) and BPA-related urinary MDA levels (Figure 5). We previously reported that the wheat sprout (*Triticum aestivum*) juice, whose main component is chlorophyll, decreases urinary BPA levels and reduces BPA-related oxidative stress [24]. The basic structure of chlorophyll is a porphyrin ring and the chemopreventive mechanism of porphyrins is suspected as trapping reactive forms of xenobiotics by nucleophilic attack [37]. As like as porphyrins, the ginsenosides of triterpen saponins in KRG can attach to aromatic rings with hydroxyl groups and create an electron rich environment that traps the ROS. Thus, our study suggests KRG inhibits BPA absorption and BPA- related oxidative stress.

Thirdly, people can suspect effects of KRG on menstrual irregularity and pain or other female-related complaints, considering phytoestrogenic structures of the ginsenosides in KRG. A case report even showed menometrorrhagia and tachyarrhythmia as side effects of ginseng [38]. However, our present results indicated KRG did not have any female-related side effects, but actually alleviated ‘menstrual irregularity’, ‘menstrual pain’, and ‘constipation’ (Figure 6). Regarding constipation in particular, a recent Japanese study showed that a ginseng-included oriental medicine, consisting of three crude drugs [processed ginger, ginseng and zanthoxylum fruit (5:3:2)], improved stool frequency and alleviated bloating and abdominal pain in patients with chronic constipation [39]. In addition, KRG improved fatigue, insomnia and depression in postmenopausal women by a decrease in cortisol/dehydroepiandrosterone sulphate ratio [40]. A recent report also showed KRG improved the quality of sleep [41]. Taken together, KRG seems to be beneficial for female-related QOL.

However, individual variation in susceptibility to KRG should be tested. Therefore, we focused on the Sasang constitutions to clarify individual variations in efficacy and QOL outcomes of KRG. In the present study, effects of KRG on anemia, flushing, and perspiration were affected by the Sasang constitutions (Figure 6). That is, the SE types showed high efficacy of KRG compared to others. These results support the recommendation that ginseng is a proper medicine for the SE type [15, 18]. Moreover, there was no one who quit the trial due to side effects of KRG or no one experienced aggravated side effects of raw ginseng, e.g. insomnia, diarrhea, headache, nosebleed, breast pain, etc., in the present study. Therefore, side effects of raw ginseng, which were observed out of Korea [42], can be related to ginseng misuse regardless consumers’ individual variations. Taken together, KRG, the processed ginseng, can be broadly used for not only the SE type but also other types with less toxicity than raw ginseng.Finally, our study includes some of study limitations. At first, some of our results were based on questionnaires, useful epidemiological tools, for end points of female symptoms. The subjects filled out the degrees of the symptoms on the questionnaires for four times during the trial. Therefore, our results should be supported by future pharmacodynamic data, e.g. blood levels of various biochemical parameters. In addition, we have a general limitation of human study, i.e. control of subjects. Although we did our best to control food-related confounders, we could not completely control all factors of food consumption. Therefore, KRG-interacted food should be clarified and considered for future study. Thirdly, we decided the period of trial as 2 weeks for sub-acute tests, considering short half-life of BPA of 12 hrs. In addition, the limited number of subjects and the outliers in the present study might induce relatively broad variation of KRG effects (Figure 4). Moreover, all humans cannot be categorized into 4 groups of the Sasang constitutions. Therefore, future longer and larger scaled trials than the present period are desired to confirm our findings.

## Conclusion

In conclusion, the present trial showed that KRG lowers urinary BPA levels and BPA-induced MDA levels and alleviates gynecological complaints without side effects of raw-ginseng. Therefore, KRG is thought to be relatively safe and chemopreventive from BPA-or female-related diseases.

## References

[1] KFDA (2011). State of the Distribution Market of Health Functional Foods.

[2] Lee HS, Park JY, Yang M (2011). Chemopreventive effects of Korean red ginseng (Panax ginseng Meyer) on exposure to PolycyclicAromatic hydrocarbons. J Ginseng Res.

[3] Nam K (2005). The comparative understanding between red ginsengs and white ginseng processed ginsengs (Panax ginseng C.A. Mayer). J Ginseng Res.

[4] Jang DJ, Lee MS, Shin BC, Lee YC, Ernst E (2008). Red ginseng for treating erectile dysfunction: a systematic review. Br J Clin Pharmacol.

[5] Hong CE, Lyu SY (2011). Anti-inflammatory and anti-oxidative effects of Korean red ginseng extract in human keratinocytes. Immune Network.

[6] Ernst E (2010). Panax ginseng: an overview of the clinical evidence. J Ginseng Res.

[7] Shim JY, Kim MH, Kim HD, Ahn JY, Yun YS, Song JY (2010). Protective action of the immunomodulator ginsan against carbon tetrachloride-induced liver injury via control of oxidative stress and the inflammatory response. Toxicol Appl Pharmacol.

[8] Kho MJ, Minji B, Yang M (2010). Chemopreventive effects of Korean red ginseng on urinary bisphenol a and malondialdehyde. Cancer Prev Res (Korean).

[9] Park Y, Kwon HY, Shimi MK, Rhyu MR, Lee Y (2011). Improved lipid profile in ovariectomized rats by red ginseng extract. Die Pharmazie.

[10] Hao K, Gong P, Sun SQ, Hao HP, Wang GJ, Dai Y, Liang Y, Xie L, Li FY (2011). Beneficial estrogen-like effects of ginsenoside Rb1, an active component of Panax ginseng, on neural 5-HT disposition and behavioral tasks in ovariectomized mice. Eur J Pharmacol.

[11] Lind PM, Eriksen EF, Lind L, Orberg J, Sahlin L (2004). Estrogen supplementationmodulates effects of the endocrine disrupting pollutant PCB126 in rat bone and uterus: diverging effects in ovariectomized and intact animals. Toxicology.

[12] Hwang SY, Kim WJ, Wee JJ, Choi JS, Kim SK (2004). Panax ginseng improves survival and sperm quality in guinea pigs exposed to 2,3,7,8- tetrachlorodibenzo- p-dioxin. BJU Int.

[13] Shim MK, Lee YJ (2012). Estrogen receptor is activated by korean red ginseng *in vitro* but not in vivo. J Ginseng Res.

[14] Kim SY, Seo SK, Choi YM, Jeon YE, Lim KJ, Cho S, Choi YS, Lee BS (2012). Effects of red ginseng supplementation on menopausal symptoms and cardiovascular risk factors in postmenopausal women: a double-blind randomized controlled trial. Menopause.

[15] Jia L, Zhao Y (2009). Current evaluation of the millennium phytomedicine–ginseng (I): etymology, pharmacognosy, phytochemistry, market and regulations. Curr Med Chem.

[16] Lee J-M (1894). Dong-Yi-Soo-Se-Bo-Won.

[17] Lee SMS, Kim H, Kim J (2007). A clinical study on the Sasang constitutional preference for foods. Korean J Oriental Med.

[18] Chae H, Lyoo IK, Lee SJ, Cho S, Bae H, Hong M, Shin M (2003). An alternative way to individualized medicine: psychological and physical traits of Sasang typology. J Altern Complement Med.

[19] Kim JY, Duong DP (2009). Sasang constitutional medicine as a holistic tailored medicine. Evid-Based Compl Alt.

[20] Theo C, Dianne D, John Peterson M (1997). A Scientific Detective Story. Our stolen future: are we threatening our fertility, intelligence, and survival?.

[21] Yang M, Kim SY, Chang SS, Lee IS, Kawamoto T (2006). Urinary concentrations of bisphenol A in relation to biomarkers of sensitivity and effect and endocrine related health effects. Environ Mol Mutagen.

[22] Sabatini L, Wilson C, Lower A, Al-Shawaf T, Grudzinskas JG (1999). Superoxide dismutase activity in human follicular fluid after controlled ovarian hyperstimulation in women undergoing in vitro fertilization. Fertil Steril.

[23] Mok-Lin E, Ehrlich S, Williams PL, Petrozza J, Wright DL, Calafat AM, Ye X, Hauser R (2010). Urinary bisphenol A concentrations and ovarian response amongwomen undergoing IVF. Int J Androl.

[24] Yi B, Kasai H, Lee HS, Kang Y, Park JY, Yang M (2011). Inhibition by wheat sprout (*Triticum aestivum*) juice of bisphenol A-induced oxidative stress in youngwomen. Mut Res.

[25] KFDA (2010): *Health Functional Food Code*. http://eng.kfda.go.kr/board/board_view.php?av_seq=17&av_pg=1&board_id=ENG_RULE&textfield=&keyfield=

[26] Kim JY, Park JY, Kang HJ, Kim OY, Lee JH (2012). Beneficial effects of Korean red ginseng on lymphocyte DNA damage, antioxidant enzyme activity, and LDL oxidation in healthy participants: a randomized, double-blind, placebo-controlled trial. Nutr J.

[27] Oh KJ, Chae MJ, Lee HS, Hong HD, Park K (2010). Effects of Korean red ginseng on sexual arousal in menopausal women: placebo-controlled, double-blind crossover clinical study. J Sex Med.

[28] Choi S, Hong J, Chi S, Jung B, Ahn K (2002). A study on the association between Sasang Constitutions (QSCCII ) and Huh’s morphological diagramming. Korean J Oriental Med.

[29] Kim H, Jang CH (2007). Sensitive determanation of eleven phenolic endocrine disrupting chemicals in human urine using gas chromatography/ mass spectrometry-selected ion monitoring. Environ Eng Res (Korean).

[30] Adewale HB, Jefferson WN, Newbold RR, Patisaul HB (2009). Neonatal bisphenol-a exposure alters rat reproductive development and ovarian morphology withoutimpairing activation of gonadotropin-releasing hormone neurons. Biol Reprod.

[31] Zama AM, Uzumcu M (2010). Epigenetic effects of endocrine-disrupting chemicals on female reproduction: an ovarian perspective. Front Neuroendocrinol.

[32] Bredhult C, Sahlin L, Olovsson M (2009). Gene expression analysis of human endometrial endothelial cells exposed to Bisphenol A. Reprod Toxicol.

[33] Titus-Ernstoff L, Troisi R, Hatch EE, Wise LA, Palmer J, Hyer M, Kaufman R, Adam E, Strohsnitter W, Noller K, Herbst AL, Gibson-Chambers J, Hartge P, Hoover RN (2006). Menstrual and reproductivecharacteristics of women whose mothers were exposed *in utero* to diethylstilbestrol (DES). Int J Epidemiol.

[34] Takeuchi TTO, Ikezuki Y, Takai Y, Taketani Y (2004). Positive relationship between androgen and the endocrine disruptor, bisphenol A, in normal women and women with ovarian dysfuction. EndocrJ.

[35] Nah WH, Park MJ, Gye MC (2011). Effects of early prepubertal exposure to bisphenol A on the onset of puberty, ovarian weights, and estrous cycle in female mice. Clin Exp Reprod Med.

[36] Anderson OS, Sant KE, Dolinoy DC (2012). Nutrition and epigenetics: an interplay of dietary methyl donors, one-carbon metabolism and DNA methylation. J Nutr Biochem.

[37] Cho YS, Hong ST, Choi KH, Chang YH, Chung AS (2000). Chemopreventive activity of porphyrin derivatives against 6-sulfooxymethylbenzo[a]pyrene mutagenicity. Asian Pac J Cancer Prev.

[38] Kabalak AA, Soyal OB, Urfalioglu A, Saracoglu F, Gogus N (2004). Menometrorrhagia and tachyarrhythmia after using oral and topical ginseng. J Womens Health (Larchmt).

[39] Horiuchi Akira NY, Tanaka N (2010). Effect of traditional Japanese medicine, Daikenchuto (TJ-100) in patients with chronic constipation. Gastroenterology Res.

[40] Tode T, Kikuchi Y, Hirata J, Kita T, Nakata H, Nagata I (1999). Effect of Korean red ginseng on psychological functions in patients with severe climacteric syndromes. Int J Gynaecol Obstet.

[41] Jeong Han H, Yun Kim H, Joon Choi J, Ahn SY, Lee SH, Oh KW, Kim SY (2013). Effects of red ginseng extract on sleeping behaviors in human volunteers. J Ethnopharmacol.

[42] Ma H, Sullivan-Halley J, Smith AW, Neuhouser ML, Alfano CM, Meeske K, George SM, McTiernan A, McKean-Cowdin R, Baumgartner KB, Ballard-Barbash R, Bernstein L (2011). Estrogenic botanical supplements, health-related quality of life, fatigue, and hormone-related symptoms in breast cancer survivors: a HEAL study report. BMC Complement Altern Med.

[CR43] The pre-publication history for this paper can be accessed here: http://www.biomedcentral.com/1472-6882/14/265/prepub

